# Genetic diagnosis of pseudomyxoma peritonei originating from mucinous borderline tumor inside an ovarian teratoma

**DOI:** 10.1186/s12920-022-01188-x

**Published:** 2022-03-07

**Authors:** Ayumi Taguchi, Hirofumi Rokutan, Katsutoshi Oda, Michihiro Tanikawa, Saki Tanimoto, Kenbun Sone, Mayuyo Mori, Tetsushi Tsuruga, Shinji Kohsaka, Kenji Tatsuno, Aya Shinozaki-Ushiku, Kiyoshi Miyagawa, Hiroyuki Mano, Hiroyuki Aburatani, Tetsuo Ushiku, Yutaka Osuga

**Affiliations:** 1 Department of Obstetrics and Gynecology, Graduate School of Medicine, The University of Tokyo, Tokyo, Japan; 2 Department of Pathology, Graduate School of Medicine, The University of Tokyo, Tokyo, Japan; 3 Division of Integrative Genomics, Graduate School of Medicine, The University of Tokyo, 7-3-1 Hongo, Bunkyo-ku, Tokyo, 113-0033 Japan; 4 Division of Cellular Signaling, National Cancer Center Research Institute, Tokyo, Japan; 5 Genome Science Division, Research Center for Advanced Science and Technology, The University of Tokyo, Tokyo, Japan; 6 Laboratory of Molecular Radiology, Center for Disease Biology and Integrative Medicine, Graduate School of Medicine, The University of Tokyo, Tokyo, Japan

**Keywords:** Pseudomyxoma peritonei, Ovarian teratoma, Mucinous borderline tumor, Comprehensive genomic profiling, Uniparental disomy, Case report

## Abstract

**Background:**

Pseudomyxoma peritonei is a rare disease condition mainly caused by primary mucinous tumors from the appendix and rarely from the ovary, such as when mucinous ovarian tumors arise from within a teratoma. Molecular analyses of pseudomyxoma from the appendix showed that *KRAS* and *GNAS* pathogenic variants are common genetic features of pseudomyxoma peritonei. However, the origin of the tumors is difficult to be identified via genetic variants alone. This study presents a case of pseudomyxoma peritonei of ovarian origin, which was diagnosed by comprehensive genomic profiling with ploidy analysis in a series of primary, recurrent, and autopsy tumor specimens.

**Case presentation:**

A 40-year-old woman was diagnosed with Stage IC2 mucinous ovarian tumor of borderline malignancy with mature cystic teratoma, upon clinical pathology. Immunohistochemical analysis suggested that the mucinous tumor was derived from the intestinal component of an ovarian teratoma. Three years later, intraperitoneal recurrence was detected, which subsequently progressed to pseudomyxoma peritonei. Genomic analysis detected *KRAS* (G12D), *GNAS* (R201C), and *FBXW7* (R367*) variants in the primary tumor. In addition, the tumor showed aneuploidy with loss of heterozygosity (LOH) in all its chromosomes, which suggested that the primary ovarian tumor was derived from germ cells. Existence of one Barr body suggested the existence of uniparental disomy of the tumors throughout the genome, instead of a haploid genotype. All three pathogenic variants remained positive in the initial recurrent tumor, as well as in the paired DNA from the whole blood in pseudomyxoma peritonei. The pathogenic variant of *KRAS* (G12D) was also identified in the autopsy specimen of the appendix by droplet digital polymerase chain reaction.

**Conclusions:**

This study pathologically and genetically confirmed that the primary ovarian borderline tumor was derived from the intestinal component of an ovarian teratoma, and that the subsequent pseudomyxoma peritonei progressed from the primary ovarian tumor. Integrative genomic analysis was useful to identify cellular origin of tumors, as well as to precisely interpret the process of disease progression.

**Supplementary Information:**

The online version contains supplementary material available at 10.1186/s12920-022-01188-x.

## Background

Comprehensive genomic profiling by targeted next-generation sequencing (NGS) panels have been prevalent in cancer precision medicine. Such panels are mainly used to identify actionable variants of genome-matched treatment options; however, the findings of single nucleotide variants, small insertions/deletions, gene fusions, tumor content ratio, and ploidy (allele-specific, chromosomal copy numbers) using NGS panels should be useful to precisely diagnose and interpret tumorigenesis in patients with cancer. A tumor-normal paired, NGS-based panel, named Todai OncoPanel (TOP) was originally developed. TOP is composed of a twin DNA and RNA panel, which covers 464 genes by the DNA panel for SNVs, In/Dels, copy number variations (CNVs), and ploidy, and 463 genes by the RNA panel for gene fusions, exon skipping, and specific gene expression [1].

Pseudomyxoma peritonei is a rare disease condition caused by primary mucinous tumors from the appendix or ovary [2]. Most of the pseudomyxoma peritonei are considered to have originated from the appendix, even if accompanied with ovarian tumor [3, 4]. If both the ovary and appendix are involved, site of origin is usually determined based on clinical, pathological, immunohistochemical, and genetic features [5]. Molecular analyses of appendiceal pseudomyxoma showed that *KRAS* and *GNAS* pathogenic variants are common genetic features of pseudomyxoma peritonei [6, 7]. However, molecular profiling of pseudomyxoma peritonei of extra-appendiceal origin has not been well described.

This study presented a case of a mucinous ovarian tumor with borderline malignancy arising from a teratoma, which subsequently developed extensive peritoneal dissemination, and eventually progressed to pseudomyxoma peritonei. TOP analyses were performed to identify the cellular origin of the mucinous borderline ovarian tumor and the subsequent pseudomyxoma peritonei to elucidate the mechanisms of disease progression.

## Case presentation

### Clinical course 1

**Fig. 1:**
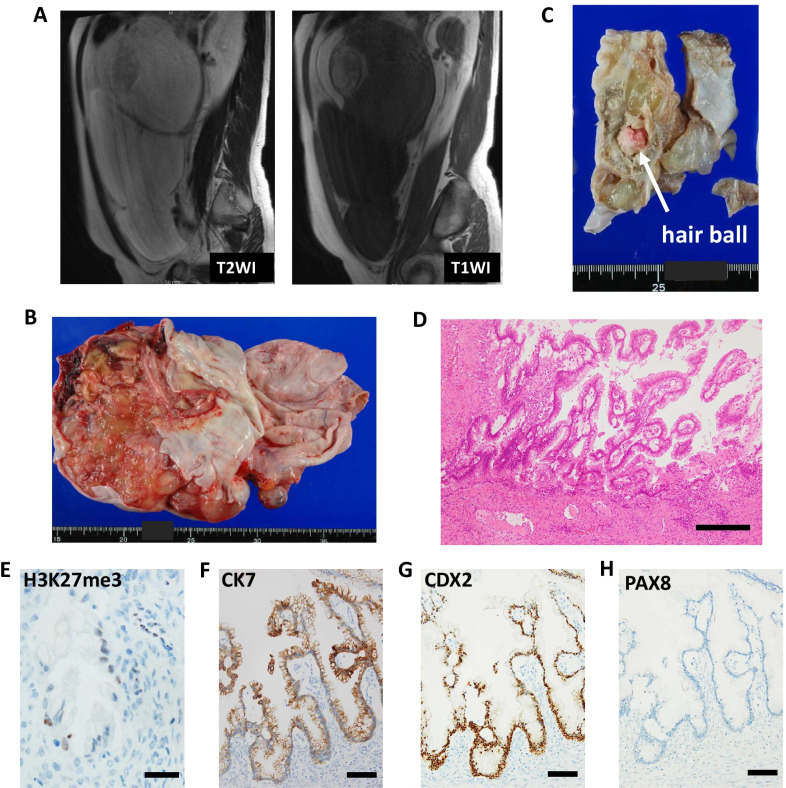
Radiologic, macroscopic, and microscopic findings at the initial surgery. **a** Magnetic resonance imaging (MRI) findings before first surgery. **b, c** Macroscopic findings of right ovarian tumor at initial surgery. **d** Hematoxylin and eosin staining (scale bar: 250 μm, × 100 magnification). Immunohistochemical analysis of **e** H3K27me3 (Barr body, scale bar: 25 μm, × 400 magnification), **f** CK7 (scale bar: 100 μm, × 200 magnification), **g** CDX2 (scale bar: 100 μm, × 200 magnification), and **h** PAX8 (scale bar: 100 μm, × 200 magnification)

A 40-year-old Japanese woman underwent a right salpingo-oophorectomy with partial omentectomy under the diagnosis of multilocular ovarian tumor, accompanied with mature cystic teratoma (Fig. 1a). The patient’s medical history was uneventful, and her family history did not suggest any hereditary cancers. The ovarian tumor spontaneously ruptured leading to excretion of mucinous fluid in the abdominal cavity. The pathological diagnosis was a Stage IC2 mucinous ovarian tumor with borderline malignancy, accompanied by a mature cystic teratoma (pT1c2NxM0, Fig. 1b–d). Immunohistochemistry revealed that the ovarian tumor was positive for CK7, CK20, and CDX2, and negative for PAX8 (Fig. 1f–h, Additional file 1), which suggested that the mucinous ovarian tumor was derived from the intestinal component of an ovarian teratoma.

**Fig. 2:**
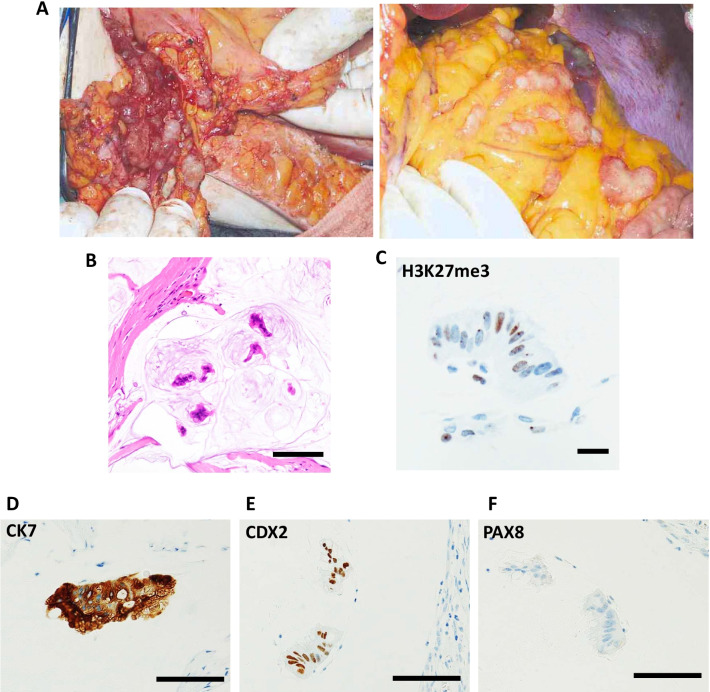
Macroscopic and microscopic findings at the secondary surgery. **a** Intra-abdominal findings at the second surgery with peritoneal dissemination (left) and omental dissemination (right). **b** Hematoxylin and eosin staining (scale bar: 250 μm, × 200 magnification). Immunohistochemical analysis of **c** H3K27me3 (Barr body, scale bar: 25 μm, × 400 magnification), **d** CK7 (scale bar: 100 μm, × 200 magnification), **e** CDX2 (scale bar: 100 μm, × 200 magnification), and **f** PAX8 (scale bar: 100 μm, × 200 magnification)

**Fig. 3:**
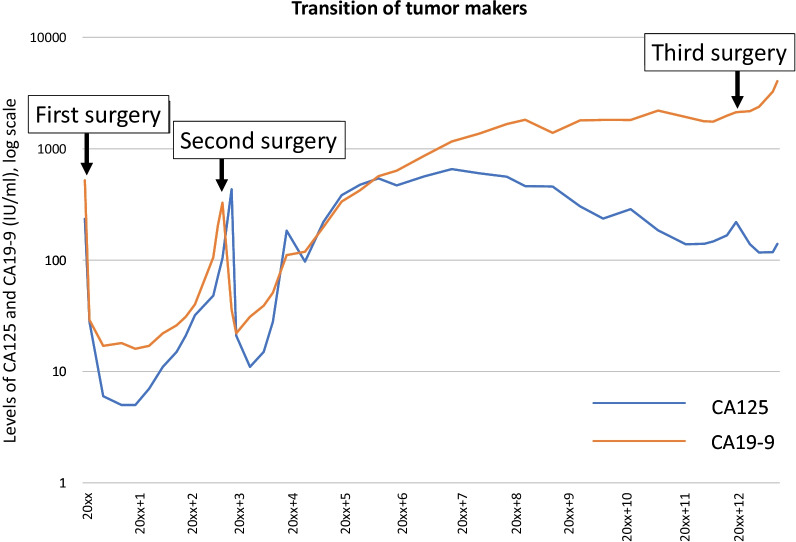
Clinical course. Change in the level of CA125 and CA19-9 during the clinical course

Three years later, initial intraperitoneal recurrence was observed, accompanied with elevated tumor markers (CA125 and CA19-9). The patient underwent subsequent cytoreductive surgery, including total hysterectomy and resection of disseminations. Surgical findings showed that peritoneal dissemination had spread diffusely throughout the peritoneal cavity with the normal-appearing appendix (Fig. 2a). Pathological findings revealed recurrent mucinous ovarian tumors (Fig. 2b, d–f). One year later, a second recurrence was observed, accompanied with elevated tumor markers and massive ascites. The patient desired conservative management without any chemotherapies or surgeries for 8 years, as it was being confirmed that the component of pseudomyxoma could be effectively discharged through urination via a spontaneous fistula between the peritoneum and the bladder. However, repeated palliative paracentesis was required for the extensive pseudomyxoma fluid from multiple isolated intraperitoneal foci (Fig. 3).

### Genetic findings of ovarian tumor and subsequent peritoneal dissemination

**Fig. 4:**
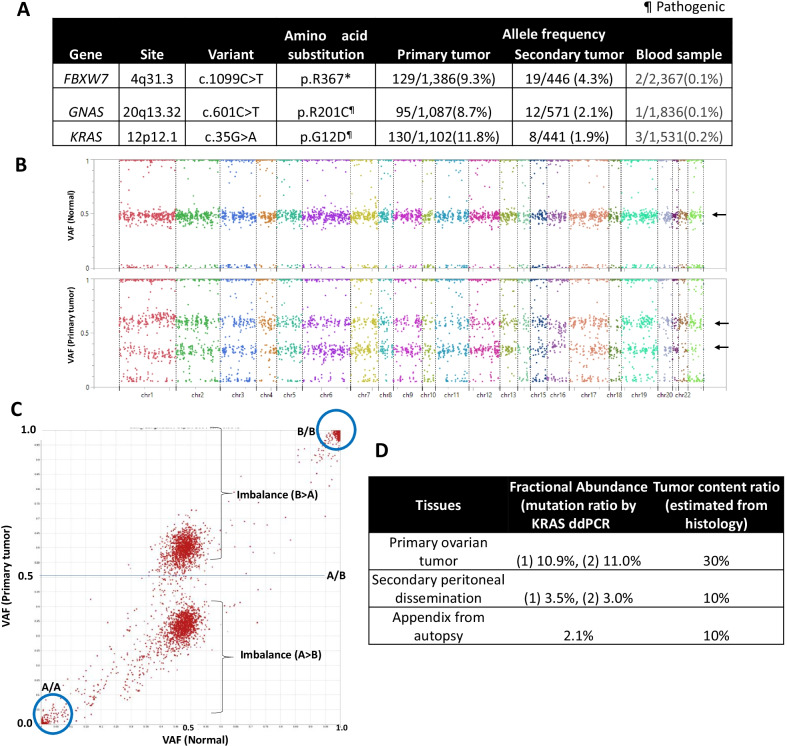
Genomic analysis. **a** Non-synonymous, pathogenic, somatic variants of the primary ovarian tumor. **b** Mapping of the variant allele frequency (VAF) of each SNP using TOP DNA panel in the normal (upper panel) and primary ovarian tumor (lower panel). Arrow: A/B hetero SNPs. **c** Distribution of the VAF of each SNP in the primary ovarian tumor and a paired normal sample. **d** Droplet digital PCR (ddPCR) of *KRAS* pathogenic variant, using the primary ovarian tumor, peritoneal dissemination at the initial recurrence, and appendix obtained from autopsy

In order to identify the genetic variants of each tumor, TOP analyses of both the primary ovarian borderline tumor and the recurrent peritoneal dissemination were performed (Additional file 1). TOP DNA panel was also used to evaluate a genome-wide view of the allele-specific copy number variants, as well as the distribution of the variant allele frequencies (VAF) of each single nucleotide polymorphism (SNP) in the primary ovarian tumor (with a paired normal sample). TOP DNA panel identified *KRAS* (G12D), *GNAS* (R201C), and *FBXW7* (R367*) variants in the primary tumor (Fig. 4a). The SNP genotypes (A/A-A/B-B/B) showed a clear imbalance of the VAF (apart from 0.5) in all the hetero (A/B) SNPs of the primary tumor sample, although not in those of the normal cells (all the A/B VAF at approximately 0.5) (Fig. 4b, c). The karyogram of the allele-specific copy number, according to the SNP genotypes, showed that the number of one allele was lower than that of the opposite allele throughout the genome (aneuploidy with LOH throughout all the chromosomes) (Additional file 2: Fig. S1). According to these findings, the primary ovarian tumor was considered to be derived from the germ cells (i.e. teratomas).

In contrast, no somatic variants with > 5% variant allele frequency (VAF) were detected in the recurrent tumor, due to the low tumor content ratio in the pseudomyxoma fluid. Therefore, it was difficult to evaluate the imbalance of the SNP genotypes in the recurrent tumor (Additional file 2: Fig. S2). However, all of the three pathogenic variants, *KRAS* (G12D), *GNAS* (R201C), and *FBXW7* (R367*) remained positive in the recurrent tumor with VAFs ranging from 1.9 to 4.3% (Fig. 4a). Therefore, pseudomyxoma peritonei was genetically diagnosed as a recurrence of the mucinous borderline ovarian tumor. In addition, these three variants were also detected in the normal-paired DNA, from the peripheral blood sample obtained during the second recurrence (from whole blood, not from plasma), with allele frequency of 0.2% for *KRAS* (G12D) and 0.1% for *GNAS* (R201C) and *FBXW7* (R367*) (Fig. 4d), which suggest either the existence of circulating tumor cells or highly abundant cell free DNA from the tumor. Because the primary ovarian tumor showed the general LOH pattern (Fig. 4b), the presence of a Barr body [8] was also investigated to distinguish between haploid (with a single set of chromosomes and no Barr body) and uniparental disomy (UPD) (i.e. one Barr body with an inactive X chromosome from the duplication of a single chromosome and LOH of the opposite allele). Both the primary ovarian tumor and the subsequent peritoneal dissemination possessed one Barr body (Figs. 1e, 2c), suggesting that both tumors showed a UPD genotype, arising from the ovarian teratoma.

### Clinical course 2 and autopsy findings

Due to disease progression, the pseudomyxoma peritonei became uncontrollable. In order to reduce dissemination, the patient received a third surgery 12 years after the first surgery. However, without sufficient and effective debulking, the patient expired due to colon perforation.

**Fig. 5:**
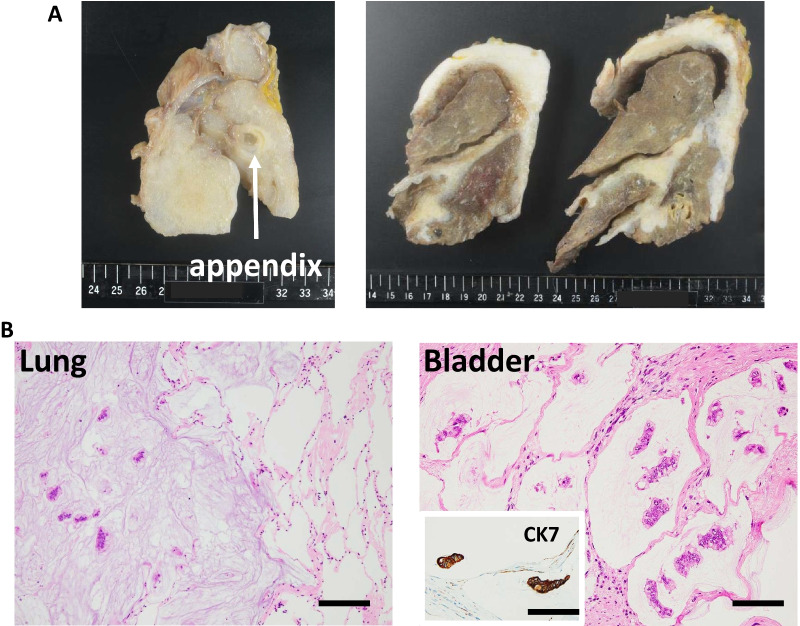
Autopsy findings. **a** Macroscopic findings of appendix (left) and lungs (right). **b** Hematoxylin and eosin staining of lung (left, scale bar: 250 μm, × 100 magnification) and bladder (right) with CK7 immunohistochemistry of bladder tumor (scale bar: 100 μm, × 200 magnification)

Informed consent for full body autopsy was taken from her family. The mucinous tumor was spread invasively to the intestine, colon, kidney, liver, bilateral diaphragm, and right lung (Fig. 5a, b). The pelvic organs including the rectum, bladder, intestine, and sigmoid colon coalesced into a single lump. Perforation was detected in the transverse colon. Microscopically, atypical mucinous epithelium was detected, which was diagnosed as recurrent mucinous borderline ovarian tumor. Intraepithelial disease was detected in the epithelium of the appendix, with massive invasion from the serosa of the appendix.

### Assessment of KRAS (G12D) in the primary ovarian tumor, peritoneal dissemination, and appendix

To confirm our molecular diagnosis where the ovary was identified as the main origin of pseudomyxoma, a detailed examination of the appendix was performed. Pathological findings alone were inconclusive in terms of identifying the tumor origin. Considering the low VAFs of the pathogenic variants of the three driver genes in the first recurrent tumor, droplet digital PCR (ddPCR) of the identified *KRAS* variant was performed (Additional file 1). ddPCR of *KRAS* variants (covering G12A, G12C, G12D, G12R, G12S, G12V, and G13D) by using the primary ovarian tumor, peritoneal dissemination at the initial recurrence, and appendix obtained from autopsy identified that all the three tumors were positive for the *KRAS* (G12D) variant with allele frequencies of 10.9–11.0% in the ovarian tumor (presumed tumor content ratio at 30% by hematoxylin and eosin staining), 3.0–3.5% in the peritoneal dissemination (presumed tumor content rate at 10%), and 2.1% in the appendiceal tumor (presumed tumor content rate at 10%), respectively (Fig. 4d). The presence of the *KRAS* (G12D) variant confirmed the metastatic nature of the tumors involving the appendix.

## Discussion and conclusion

In the current study, a case of mucinous borderline ovarian tumor arising from ovarian teratoma, which subsequently progressed to pseudomyxoma peritonei, was reported.

This case showed the time-dependent, phenotypic changes in the disease condition; it progressed from mucinous borderline ovarian tumor to multiple disseminations within a few years, and finally developed to pseudomyxoma peritonei. Epidemiologically, the possible origins of the pseudomyxoma are mainly the appendix and possibly extra-appendiceal locations, most commonly the ovary [2–5, 9, 10]. Pathological findings supported both possibilities that the subsequent pseudomyxoma originated from the primary ovarian tumor and the appendiceal tumor. Genetically, the subsequent peritoneal dissemination was diagnosed as recurrent ovarian borderline tumor with the three common somatic variants (*KRAS* (G12D), *GNAS* (R201C), and *FBXW7* (R367*)) of the primary ovarian tumor. Although tumor content ratio of the recurrent tumor was low, the common *KRAS* (G12D) variant was confirmed by ddPCR in all the samples examined. All these considered, the patient’s disease was characterized by serial progression, arising originally from the primary ovarian borderline tumor.

In this study, the mucinous borderline ovarian tumor was accompanied by a mature cystic teratoma. Cases of mucinous cystadenoma (including those with borderline malignant potential) accompanied with mature cystic teratoma are divided into two types: those derived from the intestinal mucosal components of a teratoma and collision tumors, or those arising independently from a teratoma [11, 12]. Combination of CK7, CK20, and CDX2 immunohistochemistry is usually used for the differential diagnosis of an original tumor and a collision tumor. CK7 is usually positive in collision tumors from ovarian epithelia, and strong CK20 staining and partial or negative CK7 staining usually indicates that tumors arise from the intestinal component of teratoma [13]. In this study, the tumor was strongly positive for CK7 and CK20, suggesting that immunohistochemistry alone was not sufficient to conclude the mechanism of tumorigenesis. Therefore, genetic findings of the haploid pattern (UPD genotype) in the primary mucinous borderline ovarian tumor were integral in diagnosing the germ cell origin (derived from intestinal component of teratoma) of this tumor. This is the first report that identified the origin of the pseudomyxoma peritonei from the component of mucinous borderline ovarian tumor, accompanied with teratoma, based on genetic analysis. In this case, *GNAS* and *KRAS* pathogenic variants were detected in the primary mucinous ovarian tumor. Both of gene variants were frequently detected in the lower intestinal borderline tumors. These results suggested that the genetic profile may be similar by coincidence, despite the true origins of the tumor under investigation.

There are several limitations in this study. First, the tumors involving the appendix were evaluated using the autopsy specimens, which made it difficult to pathologically discern true origin. The possibility that the appendiceal tumor itself was a primary teratoma with UPD and *KRAS* (G12D) variant should be considered, although the intraoperative findings were negative in the primary and secondary surgeries. Second, the SNP genotyping of A/A-A/B-B/B ratios was difficult in the recurrent tumor due to the low tumor content ratio. Third, VAF of the peritoneal dissemination in the TOP analysis was very low, which might overlook the key driver mutations. Lastly, the quality of DNA from FFPE specimens in the pseudomyxoma peritonei may have been low, due to long-term storage and low tumor content ratio during the long course of diseases.

Nevertheless, the comprehensive genomic profiling by TOP was useful to identify the origin of the tumors, especially by using the allele-specific copy number ‘karyogram’ to determine ploidy (Fig. 4b). Recently, extensive copy number variations gathered much attention to predict drug sensitivity, including LOH score (by FoundationOne CDx comprehensive genomic profiling) and genomic instability score (by Myriad myChoice CDx) for prediction of sensitivity to PARP inhibitors [14–16]. In addition, tumor aneuploidy correlates with markers of immune evasion and with reduced response to immunotherapy [17]. Therefore, the genome-wide evaluation of CNVs is anticipated to further enhance precision medicine for both diagnostic and therapeutic purposes.

In conclusion, this study presented a case of mucinous borderline ovarian tumor arising from the intestinal component of an ovarian teratoma, which progressed to pseudomyxoma peritonei. This study pathologically and genetically confirmed two points: (i) the primary ovarian borderline tumor was derived from the intestinal component of an ovarian teratoma with UPD genotype; and (ii) the pseudomyxoma peritonei was subsequent to serial disease progression from the primary ovarian tumor. Comprehensive genomic analysis is useful to identify the cellular origin of tumors as well as to precisely determine the stepwise process of disease progression.

## Supplementary Information

**Additional file 1**. Supplementary Materials and Methods

**Additional file 2: Figure S1**. Genome-wide view (karyogram) of the total (Upper) and allele-specific copy number variants (Lower) in the primary ovarian tumor using the TOP DNA panel, constructed with the hetero SNP genotypes between the primary tumor and paired-normal cells. The Y-axis reflects the log2 copy number ratios. The copy number of one allele (blue line) is lower than that of the opposite allele (red line) throughout the genome, which suggests the existence of a genome-wide LOH. The baseline copy number of red line (Y-axis) cannot be determined using this data alone. **Figure S2**. Mapping of the VAF of each SNP in a recurrent tumor sample. (The imbalance of hetero A/B SNP ratio is severely affected when the tumor content ratio is low.)

## Data Availability

The sequencing data of TOP analysis in this case have been deposited to the MGeND database (Medical Genomics Japan Variant Database, https://mgend.med.kyoto-u.ac.jp/). In addition, the datasets are available in the NBDC Human Database (https://humandbs.biosciencedbc.jp/en/) with the accession numbers hum0157, and the raw datasets are available in the DDBJ (https://ddbj.nig.ac.jp/search) with the accession numbers (jga-study > JGAS000164and jga-dataset > JGAD000242). The patient’ ID is 000043-01 (for the primary tumor) and 000043-02 (for the recurrent tumor).

## Abbreviations

CNVs: Copy number variations
ddPCR: Droplet digital PCR
LOH: Loss of heterozygosity
NGS: Next-generation sequencing
UPD: Uniparental disomy

## References

[1] Kohsaka S, Tatsuno K, Ueno T, Nagano M, Shinozaki-Ushiku A, Ushiku T, Takai D, Ikegami M, Kobayashi H, Kage H, Ando M, Hata K, Ueda H, Yamamoto S, Kojima S, Oseto K, Akaike K, Suehara Y, Hayashi T, Saito T, Takahashi F, Takahashi K, Takamochi K, Suzuki K, Nagayama S, Oda Y, Mimori K, Ishihara S, Yatomi Y, Nagase T, Nakajima J, Tanaka S, Fukayama M, Oda K, Nangaku M, Miyazono K, Miyagawa K, Aburatani H, Mano H (2019). Comprehensive assay for the molecular profiling of cancer by target enrichment from formalin-fixed paraffin-embedded specimens. Cancer Sci.

[2] Moran BJ, Cecil TD (2003). The etiology, clinical presentation, and management of pseudomyxoma peritonei. Surg Oncol Clin N Am.

[3] Young RH, Gilks CB, Scully RE (1991). Mucinous tumors of the appendix associated with mucinous tumors of the ovary and pseudomyxoma peritonei. A clinicopathological analysis of 22 cases supporting an origin in the appendix. Am J Surg Pathol.

[4] Prayson RA, Hart WR, Petras RE (1994). Pseudomyxoma peritonei. A clinicopathologic study of 19 cases with emphasis on site of origin and nature of associated ovarian tumors. Am J Surg Pathol.

[5] Chuaqui RF, Zhuang Z, Emmert-Buck MR, Bryant BR, Nogales F, Tavassoli FA, Merino MJ (1996). Genetic analysis of synchronous mucinous tumors of the ovary and appendix. Hum Pathol.

[6] Noguchi R, Yano H, Gohda Y, Suda R, Igari T, Ohta Y, Yamashita N, Yamaguchi K, Terakado Y, Ikenoue T, Furukawa Y (2015). Molecular profiles of high-grade and low-grade pseudomyxoma peritonei. Cancer Med.

[7] Nummela P, Saarinen L, Thiel A, Järvinen P, Lehtonen R, Lepistö A, Järvinen H, Aaltonen LA, Hautaniemi S, Ristimäki A (2015). Genomic profile of pseudomyxoma peritonei analyzed using next-generation sequencing and immunohistochemistry. Int J Cancer.

[8] Barr ML, Bertram EG (1949). A morphological distinction between neurons of the male and female, and the behaviour of the nucleolar satellite during accelerated nucleoprotein synthesis. Nature.

[9] Pranesh N, Menasce LP, Wilson MS, O'Dwyer ST (2005). Pseudomyxoma peritonei: unusual origin from an ovarian mature cystic teratoma. J Clin Pathol.

[10] Yan F, Shi F, Li X, Yu C, Lin Y, Li Y, Jin M (2020). Clinicopathological characteristics of pseudomyxoma peritonei originated from ovaries. Cancer Manag Res.

[11] Ueda G, Fujita M, Ogawa H, Sawada M, Inoue M, Tanizawa O (1993). Adenocarcinoma in a benign cystic teratoma of the ovary: report of a case with a long survival period. Gynecol Oncol.

[12] Roy S, Mukhopadhayay S, Gupta M, Chandramohan A (2016). Mature cystic teratoma with co-existent mucinous cystadenocarcinoma in the same ovary—a diagnostic dilemma. J Clin Diagn Res.

[13] Clark ME, Will MD (2016). Intestinal-type adenocarcinoma arising in a mature cystic teratoma of the ovary. Int J Gynecol Pathol.

[14] Coleman RL, Oza AM, Lorusso D, Aghajanian C, Oaknin A, Dean A, Colombo N, Weberpals JI, Clamp A, Scambia G, Leary A, Holloway RW, Gancedo MA, Fong PC, Goh JC, O'Malley DM, Armstrong DK, Garcia-Donas J, Swisher EM, Floquet A, Konecny GE, McNeish IA, Scott CL, Cameron T, Maloney L, Isaacson J, Goble S, Grace C, Harding TC, Raponi M, Sun J, Lin KK, Giordano H, Ledermann JA (2017). Rucaparib maintenance treatment for recurrent ovarian carcinoma after response to platinum therapy (ARIEL3): a randomised, double-blind, placebo-controlled, phase 3 trial. Lancet.

[15] González-Martín A, Pothuri B, Vergote I, DePont CR, Graybill W, Mirza MR, McCormick C, Lorusso D, Hoskins P, Freyer G, Baumann K, Jardon K, Redondo A, Moore RG, Vulsteke C, O'Cearbhaill RE, Lund B, Backes F, Barretina-Ginesta P, Haggerty AF, Rubio-Pérez MJ, Shahin MS, Mangili G, Bradley WH, Bruchim I, Sun K, Malinowska IA, Li Y, Gupta D, Monk BJ (2019). Niraparib in patients with newly diagnosed advanced ovarian cancer. N Engl J Med.

[16] Ray-Coquard I, Pautier P, Pignata S, Pérol D, González-Martín A, Berger R, Fujiwara K, Vergote I, Colombo N, Mäenpää J, Selle F, Sehouli J, Lorusso D, Guerra Alía EM, Reinthaller A, Nagao S, Lefeuvre-Plesse C, Canzler U, Scambia G, Lortholary A, Marmé F, Combe P, de Gregorio N, Rodrigues M, Buderath P, Dubot C, Burges A, You B, Pujade-Lauraine E, Harter P (2019). Olaparib plus bevacizumab as first-line maintenance in ovarian cancer. N Engl J Med.

[17] Davoli T, Uno H, Wooten EC, Elledge SJ (2017). Tumor aneuploidy correlates with markers of immune evasion and with reduced response to immunotherapy. Science.

