# Supplementary material for: Genetic diagnosis of pseudomyxoma peritonei originating from mucinous borderline tumor inside an ovarian teratoma
Source: BMC Med Genomics. 2022 Mar 7;15:51. doi: 10.1186/s12920-022-01188-x (PMC8900394; doi:10.1186/s12920-022-01188-x)
Supplement: Supplementary file 1 — Additional file 1. Supplementary Materials and Methods [file 12920_2022_1188_MOESM1_ESM.docx]

**Supplementary Information**

**Materials and Methods**

**The Patient**

Written informed consent was obtained from the patient and the next of kin. This study was approved by the Institutional Ethics Committee of the University of Tokyo (Approval number: G10114-(15). The clinical information was obtained from electronic medical records.

**Immunohistochemistry**

The immunohistochemical tests for H3K27me3, CK7, CDX2, and PAX8 were performed as follows: The surgical samples were fixed with formalin and embedded in paraffin. Four micrometer-thick sections were stained using a Ventana Benchmark XT stainer (Ventana Medical Systems, Tucson, AZ, USA). Briefly, the sections were deparaffinized and pretreated with a CC1 buffer or protease and incubated with primary antibodies, and then detected chromogenically using the iView universal 3,30-diaminobenzidine detection kits (Ventana Medical Systems). Antibody information and detailed procedures of immunohistochemistry are summarized in Supplementary Table 1. Histological and immunohistochemical images were examined and captured on a BX51 microscope (Olympus Corporation, Tokyo, Japan) connected to a DP2-SAL (Olympus) camera controller, using accessory objective lens (10x, 20x or 40x).

**Table S1 List of antibodies used in immunohistochemistry**

RTU, ready-to-use

**Comprehensive genomic profiling by TOP, a targeted next-generation sequencing panel**

Genomic DNA and total RNA were extracted from both the primary ovarian tumor and secondary peritoneal dissemination using formalin-fixed paraffin-embedded tumor tissues. Genomic DNA was also extracted from the paired peripheral blood lymphocytes. All the DNA and cDNA (from the total RNA) samples were subjected to the targeted next-generation sequencing, TOP, as described previously [1]. Briefly, the TOP DNA panel covered 464 genes for single nucleotide variants, insertions/deletions and chromosomal copy number variants, and the TOP RNA panel covered 463 genes for gene fusions, exon skipping, and specific gene expressions [1]. Somatic mutations were identified using Karkinos (<https://github.com/genome-rcast/karkinos>), which detects SNV, short indels, chromosomal allele-specific copy number variation (CNV), and tumor purity.

**Evaluation of allelic imbalances and allele-specific copy number variants using the TOP DNA panel**

The TOP DNA panel targeted 464 genes and > 4,300 single nucleotide polymorphisms (SNPs) throughout the genomes, including the introns of the targeted genes. Thus, the TOP DNA panel (with normal-paired DNA) can be used as an SNP-array, evaluating both the copy number variants and A/A-A/B-B/B (SNPs) ratios. The variant allele frequency (VAF) of the deep (> 500) read sequence depth positions were plotted along with the genomic positions, and the allelic imbalance was determined by the discrepancies in the VAF of the hetero SNPs in normal sample. The ratio of hetero A/B SNPs is theoretically 0.5 without any gains and losses, and the ratio varies to between 0–0.5 or 0.5–1.0 (according to the tumor content ratio) in samples with copy number variants, including LOH.

**Droplet digital PCR for screening of *KRAS* mutation**

Genomic DNA was extracted from the macro-dissected FFPE sections using a QIAamp DNA Micro Kit (Qiagen GmbH, Hilden, Germany). Detection of *KRAS* mutation was conducted using a QX200 Droplet Digital PCR system (ddPCR; Bio-Rad Laboratories, Hercules, CA). The samples were screened using the ddPCR *KRAS* Screening Multiplex Kit (Bio-Rad) for the presence of seven *KRAS* mutations (G12A/C/D/R/S/V and G13D) and one specific wild-type sequence within exon 2, as previously described [2]. The TaqMan PCR mixtures were assembled using 10 μL 2 × ddPCR Supermix for Probes (Bio-Rad), 1 μL 20 × multiplex primers/probes (FAM + HEX), and 10 μL of the DNA sample (100 ng) /water. The final-volume reactions of 20 μL were loaded into sample wells of a DG8 cartridge (Bio-Rad) with 70 μL of Droplet Generation Oil for Probes (Bio-Rad). The droplets were generated using the QX200 Droplet Generator (Bio-Rad); 40 μL of the generated droplets was manually transferred with a multichannel pipette into a 96-well PCR plate and amplified in a C1000 Touch thermal cycler (Bio-Rad). The thermal cycling conditions were as follows: 95°C for 10 min, 40 cycles at 94°C for 30 s, then at 55°C for 1 min, followed by 98°C for 10 min and cooling to 4°C. The droplets were analyzed using the QX200 Droplet Reader (Bio-Rad). The data analysis was performed using the QuantaSoft version 1.7.4.0917 (Bio-Rad), which uses the number of positive and negative droplets to calculate the concentration of the target and reference DNA sequences and their Poisson-based 95% CIs.

**References**

[1] Kohsaka S, Tatsuno K, Ueno T, Nagano M, Shinozaki-Ushiku A, Ushiku T, Takai D, Ikegami M, Kobayashi H, Kage H, Ando M, Hata K, Ueda H, Yamamoto S, Kojima S, Oseto K, Akaike K, Suehara Y, Hayashi T, Saito T, Takahashi F, Takahashi K, Takamochi K, Suzuki K, Nagayama S, Oda Y, Mimori K, Ishihara S, Yatomi Y, Nagase T, Nakajima J, Tanaka S, Fukayama M, Oda K, Nangaku M, Miyazono K, Miyagawa K, Aburatani H, Mano H. Comprehensive assay for the molecular profiling of cancer by target enrichment from formalin-fixed paraffin-embedded specimens Cancer Sci. 2019;110:1464-79.

[2] Ushiku T, Kunita A, Kuroda R, Shinozaki-Ushiku A, Yamazawa S, Tsuji Y, Fujishiro M, Fukayama M. Oxyntic gland neoplasm of the stomach: expanding the spectrum and proposal of terminology. Mod Pathol 2020;33:206-216.
