# Supplementary material for: Genetic diagnosis of pseudomyxoma peritonei originating from mucinous borderline tumor inside an ovarian teratoma
Source: BMC Med Genomics. 2022 Mar 7;15:51. doi: 10.1186/s12920-022-01188-x (PMC8900394; doi:10.1186/s12920-022-01188-x)
Supplement: Supplementary file 2 — Additional file 2: Figure S1. Genome-wide view (karyogram) of the total (Upper) and allele-specific copy number variants (Lower) in the primary ovarian tumor using the TOP DNA panel, constructed with the hetero SNP genotypes between the primary tumor and paired-normal cells. The Y-axis reflects the log2 copy number ratios. The copy number of one allele (blue line) is lower than that of the opposite allele (red line) throughout the genome, which suggests the existence of a genome-wide LOH. The baseline copy number of red line (Y-axis) cannot be determined using this data alone. Figure S2. Mapping of the VAF of each SNP in a recurrent tumor sample. (The imbalance of hetero A/B SNP ratio is severely affected when the tumor content ratio is low.) [file 12920_2022_1188_MOESM2_ESM.pptx]

## Slide 1
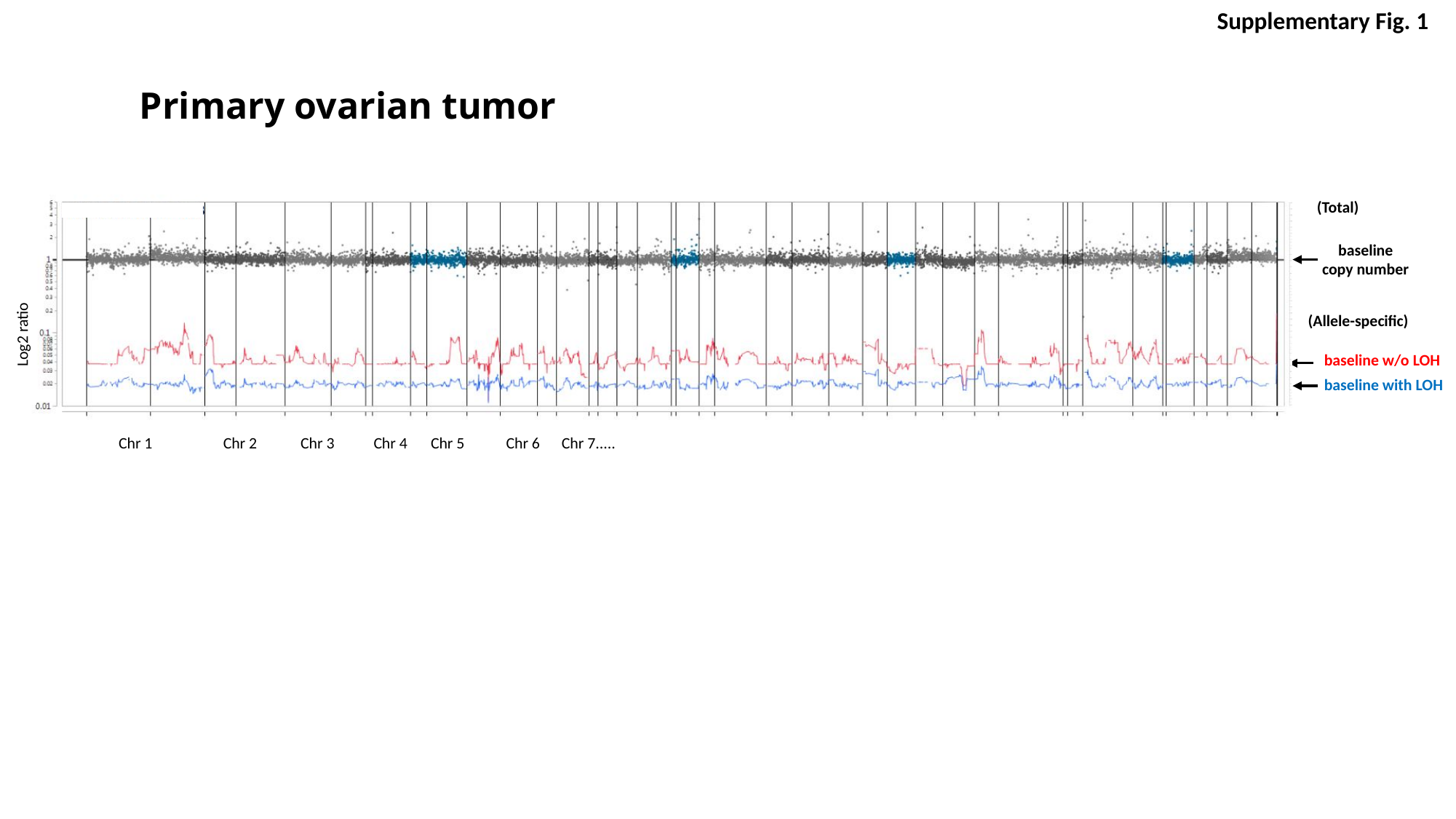

Supplementary Fig. 1
Primary ovarian tumor
(Total)
baseline
copy number
 Log2 ratio
(Allele-specific)
baseline w/o LOH
baseline with LOH
 Chr 1　　　　Chr 2　　 Chr 3　　Chr 4　Chr 5 　Chr 6 Chr 7.....

## Slide 2
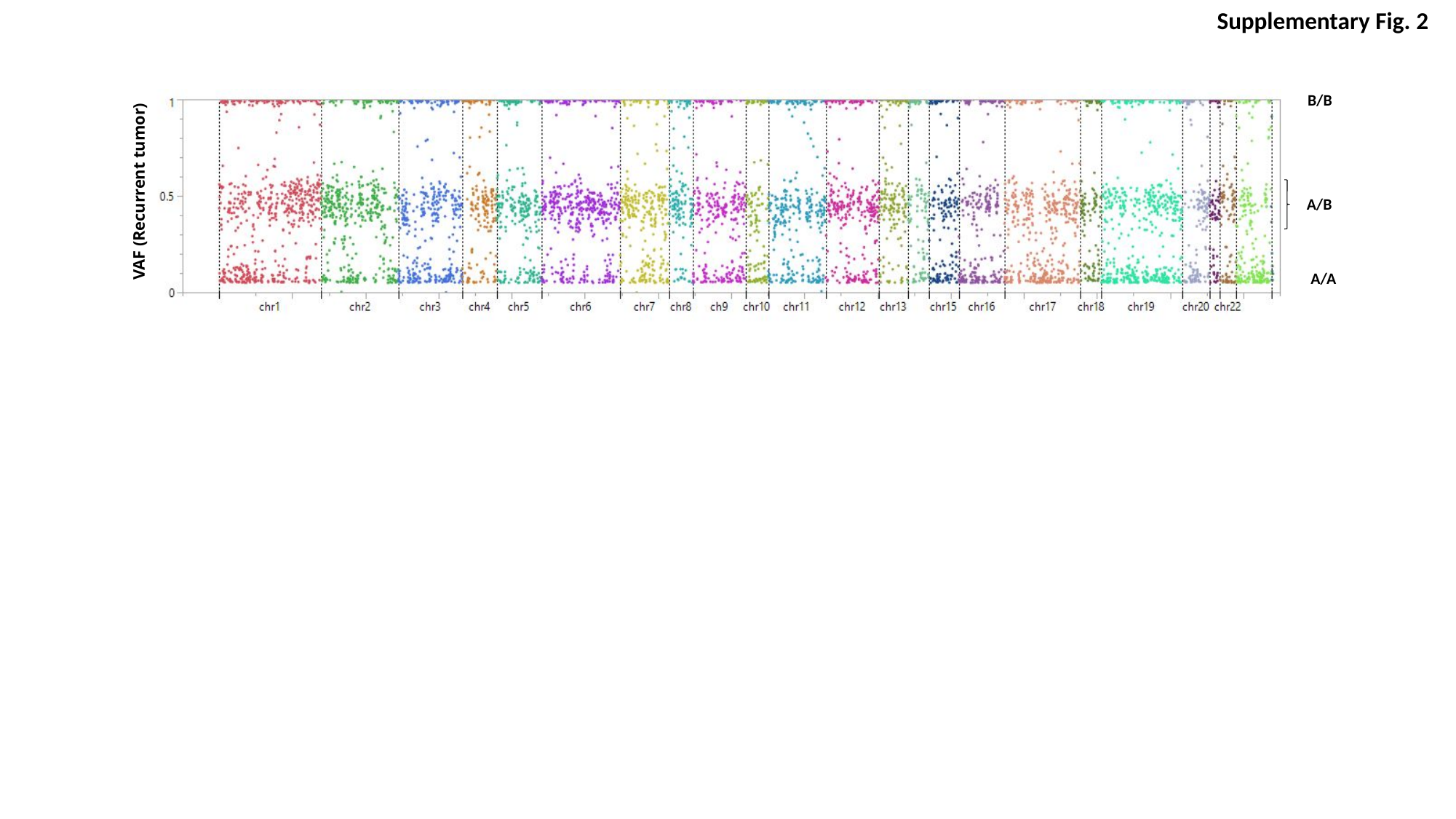

Supplementary Fig. 2
B/B
VAF (Recurrent tumor)
A/B
A/A
